# Respected but stigmatized: Healthcare workers caring for COVID-19 patients

**DOI:** 10.1371/journal.pone.0288609

**Published:** 2023-07-21

**Authors:** Ineke Spruijt, Anne Cronin, Frances Udeorji, Mamoona Nazir, Samaila Shehu, Sebastien Poix, Andre Villanueva, Niesje Jansen, Ineke Huitema, Jeanine Suurmond, Kathy Fiekert

**Affiliations:** 1 KNCV Tuberculosis Foundation, The Hague, The Netherlands; 2 KNCV Tuberculosis Foundation, Manila, The Philippines; 3 Department Social Science, Amsterdam Public Health Institute, University Medical Centre Amsterdam, Amsterdam, The Netherlands; Chulalongkorn University Faculty of Medicine, THAILAND

## Abstract

**Background:**

Healthcare workers (HCWs) caring for Corona Virus Disease 2019 (COVID-19) patients are at increased risk of being stigmatized, which compromises their individual mental well-being and the quality of care they deliver. Stigma-reduction interventions may (partly) prevent this. However, there is a lack of in-depth understanding of the experiences and underlying causes of COVID-19 stigma among HCWs, which is needed to design such interventions. We conducted in-depth semi-structured interviews to assess COVID-19 stigma among COVID-19 HCWs in Ireland, Nigeria, The Netherlands, Pakistan, and The Philippines.

**Methods:**

We used a purposive and snowball sampling to recruit a total of 53 HCWs for online interviews (13 in Ireland; 15 in Nigeria; 6 in The Netherlands; 6 in Pakistan; and 13 in The Philippines (2021). After verbatim transcribing interviews, we used a thematic approach for data analysis.

**Results:**

In all countries, stigmatization of COVID-19 HCWs is driven by fear of infection and the perception of HCWs being carriers of the disease amplified by them wearing of scrubs and personal protective equipment. There were differences between countries in the way stigma manifested in self- anticipated and experienced stigma like scolding, discrimination, avoidance, (self-) isolation, and exclusion in public, in the community, at work, and in the household. The stigma resulted in feelings of depression, loneliness, isolation, and the desire to quit one’s job.

**Discussion:**

COVID-19 HCWs from all countries experienced all forms of stigmatization related to their work as a COVID-19 frontliner. This affected their mental well-being, which in turn affects job performance and quality of care, there is a high need to develop stigma reduction tools for HCWs.

## Introduction

Despite their crucial role in fighting the Corona Virus Disease 2019 (COVID-19) pandemic by caring for patients, healthcare workers (HCWs), such as nurse-assistants, nurses, physicians, infection prevention control staff, and facility staff, are no free from stigmatization [1]. For example, HCWs in the Philippines experienced insulting gestures, physical/social loathing, social media bashing and offensive jokes [2]. In Zimbabwe, COVID-19 HCWs were avoided and shunned by their colleagues after returning to work from sick leave for COVID-19. Furthermore, other Zimbabwean COVID-19 HCWs were requested to vacate their house after neighbours and the landlord found out they were sick with COVID-19 [3]. Vietnamese HCWs were avoided by some colleagues at work (experienced stigma), feared infecting family members (self-stigma), and family members and children were labelled “Corona lady”, “Corona-children” (secondary stigma) [4].

Stigma is a social process that is characterized by exclusion, rejection, blame or devaluation which is a result of an experience, perception or anticipation of an adverse social judgement about a health condition, person or group of persons [5]. Different forms of stigma exist: 1) experienced stigma (lived experience of stigma); 2) self-stigma (a person’s own adoption of negative societal beliefs, feelings, and devaluation, associated with their stigmatized status); 3) perceived stigma (perceptions about how stigmatized groups are treated in a given context); 4) anticipated stigma (expected stigmatization by others if one’s health condition becomes known) [6]. During the stigmatization process, drivers (inherently negative, such as fear of infection, social judgment, and blame) and facilitators (either positive or negative influences, such as personal protective equipment (PPE) that can minimize or exacerbate stigmatizing avoidance behaviours) of stigma determine if stigma is applied to people or groups according to a specific health condition (stigma marking). Consequently, stigma manifests in a range of stigma experiences and practices which influence outcomes for stigmatized populations, such as access to healthcare services, uptake of testing, and adherence to treatment [6].

Stigma may fail to protect the public health as it can be an important driver of problematic behaviours that counteract the fight against an infectious disease [7]. For example, persons with infectious diseases may hide their illness and avoid seeking care out of fear of stigma. This poses a threat to public health as early identification and treatment of persons with infectious diseases, such as COVID-19, are crucial to contain the disease, protecting the wider public as well as improving the chances of recovery for the affected individual. In light of stigmatization of HCWs, it can impact the mental health of HCWs [8, 9] and can lead to elevated risk for anxiety, depression, post-traumatic stress disorder (PTSD) and even suicide [10, 11]. Compromised mental wellbeing affects job satisfaction and performance and consequently quality and quantity of patient care [12].

Effective tools and interventions are therefore needed to reduce COVID-19 stigma [13–15] and promote mental wellbeing [16]. The majority of available evidence on stigma among COVID-19 HCWs showed that perceived, anticipated, and experienced stigma occur among COVID-19 HCWs. However, these studies do not provide evidence on the underlying causes (drivers and facilitators) of COVID-19 stigma among HCWs. These insights are required to develop new or adjust existing stigma-reduction tools targeted at HCWs. Therefore, we conducted a qualitative study to assess drivers, facilitators, and manifestations of COVID-19 stigma among COVID-19 frontline HCWs in 2 high-income countries (Ireland, The Netherlands) and 3 low/middle income countries (Nigeria, Pakistan, and The Philippines).

## Materials and methods

### Study setting and population

We conducted a qualitative study (2021–2022) using online semi-structured interviews between April and September 2021 to assess the mental well-being and stigmatization of HCWs working directly with COVID-19 patients in private or public hospitals in Ireland, Nigeria, The Netherlands, Pakistan, and The Philippines. We selected these countries based on the presence of a country office of KNCV Tuberculosis Foundation and/or established contacts of the participating research assistants. Furthermore, we selected these countries for geographical diversity and their wide variety of cultural and social norms and healthcare systems. With this selection, we aimed to capture different forms of stigma.

We used purposive and snowball sampling strategies to remotely recruit participants. We chose this strategy as we expected difficulties in the number of HCWs available and willing to participate in the interviews and still wanted to ensure maximum variability in the participants (age and profession). The following authors (research assistants) recruited participants via their social contacts or country offices of KNCV Tuberculosis Foundation and coordinated interviews in the following countries: AC and FU in Ireland; FU in Nigeria; MN in Pakistan; IS in The Netherlands; and AV and SP in The Philippines. Participants to be approached were selected based on being a frontline COVID-19 HCW for multiple months in one of the selected countries. The authors approached participants by phone or email to explain study purposes, interview processes, voluntary nature of participation and confidentiality. Additionally, potential participants were provided with a project informed consent form, including the wish to audiotape the interview, and asked for their willingness to participate in the interview. After receiving a signed informed consent form, an interview date was planned. Participants chose the digital application for the interview to be conducted on: Teams, Zoom, or Google Meet. Participants received reimbursement for their participation in their local currency, equivalent to 25 Euros.

### Data collection

Author IS designed the interview topic guide and based the themes and questions on topic guides from various studies [17, 18], stigma measurement tool [19], and the health stigma and discrimination framework [6]. Authors KF, AC, FU, MN, SP, and SS discussed and adjusted the topic guide (S1 File).

After receiving training on interview skills, informed consent procedure, and the topic guide (by IS), AC, FU, MN, SS, SP conducted the interviews. Immediately following the interviews, the interviewers wrote an interview report and transcribed the interviews verbatim. In weekly online meetings, the interviewers discussed the interviews with IS and KF and identified any missed opportunities or the need to adjust the topic guide. No adjustments to the topic guide were made during this process.

We anticipated to reach data saturation per country around 15–20 semi-structured interviews. We reviewed interview data after 5 and 10 interviews to assess if data saturation had occurred or if more interviews were needed. Data saturation was reached after 13 interviews in Ireland, 14 in Nigeria, and 13 in The Philippines. Due to time constrains and difficulties to recruit participants, we conducted 6 interviews in The Netherlands and 6 in Pakistan. All interviews were conducted in English, except for the Dutch interviews in The Netherlands, and had an average duration of 39 minutes (minimum of 10 and maximum of 65 minutes). We excluded one Nigerian interview from analysis: we considered this interview to be an outliner as the interviewee was a case manager and the interview was cut short for unknown reasons to 10 minutes which we deemed too short to be able to provide in-depth answers on the topics of the interview topic list.

### Data analysis

We first familiarized ourselves with the transcripts. Consequently, we allowed for themes and subthemes to emerge by using an applied thematic approach for data analysis [20]. The identified themes and subthemes in the first ten interviews were used to develop a coding scheme to guide the coding of the remaining interviews. Author IS refined the coding scheme along the coding process. Authors IS, JS, IH, NJ, and KF discussed the interpretation of data. We used NVivo software for data analysis.

### Ethics

The Medical Ethical Committee (METC) of the Academic Medical Centre Amsterdam (AMC) waived the need for full ethical approval. All interviewees received verbal and written information about the study, the confidentiality and anonymity of personal data use and analysis, and the right to withdraw from the study at any time. Consequently, all interviewees gave informed consent for audiotaping and analysis of interviews. We followed the ethical principles of the Declaration of Helsinki, adopted by the World Medical Association [21].

## Results

### Participant characteristics

In total, we conducted 52 interviews. We interviewed 32 females and 21 males between the age of 23 and 64 years (on average 31 years). Interviewees from the Philippines were on average 43 years old and thus older compared to the other countries (average ranging from 27–33). Interviewees worked as nurse assistants (n = 9), nurses (n = 25), or physicians (n = 18) on COVID-19 departments in urban (n = 45) or rural (n = 3) hospitals. In Pakistan, we did not succeed in recruiting nurses for the interviews and only interviewed physicians, whereas in Ireland we did not succeed to recruit physicians and only interviewed nurses. Table 1 provides a detailed overview of characteristics of the interviewees and interviews.

**Table 1:** Characteristics of interviews and interview participants.

	Total	Ireland	Nigeria	The Netherlands	Pakistan	The Philippines
**Number of interviews**	52	13	14	6	6	13
**Interview characteristics**						
**Language**	-	English	English	Dutch	English	English
Length (minutes) • Average • Min. • Max.	39 26 53	38 23 60	31 10 40	45 37 50	36 32 52	51 26 65
**Age range** • Average • Min. • Max.	33 22 64	33 24 44	33 22 39	29 23 33	27 26 28	43 27 64
**Sex** • Female • Male	31 21	9 4	7 7	6 0	3 3	6 7
**Job title** • Nurse assistant • Nurse • Physician	9 25 18	6 7 0	1 11 2	1 3 2	0 0 6	1 4 8
**Hospital location** • Urban • Rural • Missing	44 3 5	6 2 5	14 0 0	5 1 0	6 0 0	13 0 0

### Experienced stigma

Non-COVID-19 HCWs, persons in public spaces, and persons in the social circle perceived COVID-19 HCWs to be carriers of the virus. This consequently led to fear (driver of stigma) to get infected with COVID-19 by COVID-19 HCWs.

*Pakistan participant 1: “I think they [non-COVID-19 HCW] are afraid of us somehow because we have been working in the emergency Department*. *They think we may have the disease.”*

Participants from all countries expressed stigmatization of non-COVID-19 HCWs within their healthcare facilities, which was experienced more often by participants from Nigeria, The Philippines, Ireland, and Pakistan then The Netherlands. Stigmatization was facilitated by the fact that participants were recognized as COVID-19 HCWs by other non-COVID-19 HCWs because of their scrubs/outfits or else because they were acquainted. Stigmatization was mostly manifested in avoiding [all countries] or scolding [Nigeria and Philippines] COVID-19 HCWs. For example, they were required to wait outside hospital pharmacies (for example when picking up medication) and manager’s and professor’s offices because they worked in the COVID-19 wards. Additionally, non-COVID-19 HCWs would take clear measures down the corridors, canteens, or hallways to avoid COVID-19 HCWs, such as turning around or taking a detour.

*Philippine participant 11*: *“So many times, I go to the pharmacy and instead of letting me in, they will make me stand far away from their entrance door and beg me not to touch the doorknob before they supply me with what I need because they do not want to come in contact with me.”*

Another manifestation of stigma was the denial of entry to other departments [Pakistan, Philippines, Nigeria]. Some COVID-19 physicians from The Philippines and Pakistan were not allowed to enter other departments to care for other patients. Some Nigerian nurses were not allowed back to their original department after completing their time at the COVID-19 department. Few Nigerian participants explained that colleagues gossiped about those who were selected to work at COVID-19 departments: COVID-19 HCWs were supposedly selected because they were young, unmarried, wanted extra payment (which was a false assumption). or else were survivors of COVID-19. This made them feel ungrateful and disrespected: they volunteered to work as COVID-19 HCWs to contribute to the fight against COVID-19. Additionally, some HCWs from Ireland, The Netherlands, and The Philippines described the avoidance of managers, supporting staff or non-COVID HCWs (such as neurologists and pain management teams) to enter the COVID-19 wards.

*Dutch participant 6: “I noticed that… a lot of people wanted badly to stay away from the [COVID-19] ward… Normally, many physicians from other disciplines would stop by to attend to patients, but now everything was done over the phone. […] At some point we though, is this because you have to put full PPE on or because you are afraid?*

*Irish participant 13: “Because in the first phase like even, like all the …medical teams reviewing patients, it was all done remotely, even palliative care weren’t coming to the wards and we had maybe 14 or 15 people dying at the one time, you know. And like, and we find that really very isolating.”*

Furthermore, some participants from Nigeria, the Philippines and Pakistan were scolded by non-COVID-19 HCWs. They were called “Dr. Coro is here”; “infectious”; and “Hey COVID”. One Nigerian participant expressed that the nickname calling was no different from her previous position in an infectious disease ward (intersecting stigma).

*Nigerian participant 11: “They are still running away. Even before COVID-19 virus came, they were calling me infectious Nurse because I worked in the infectious unit when there was Lassa fever.”*

Some Nigerian participants were stigmatized and harassed by their patients. One participant working on ambulance services and wearing PPE experienced a patient refusing care because he/she did not want to be associated with COVID-19 HCWs. Another participant explained the harassment from some patients faced while on duty.

*Nigerian participant 12: “We could go to a patient who is a suspected case, and they will remove their mask and cough in our faces. […] We beg them to use their masks when we are in their rooms, and they feel like we are ridiculing them. But then when their family pays them a visit, they will not want us to let them in, for fear of infecting them. But why would they think US healthcare workers our lives are less than that of their family […] Some of them had terrible attitudes.”*

Almost all participants experienced some type of stigmatization from their friends and/or family and in public [all countries]. The stigma was driven by fear of infection and lack of knowledge on risks of infection. Participants, particularly in the Philippines and Nigeria, also perceived misinformation about COVID-19 spread by the media and the government as a driver of stigma which created fear of infection by the public. A facilitator of the experienced stigma was the PPE that participants would wear, such as scrubs and N95 facemasks, and by which the public could identify them as HCWs. Wearing scrubs in public is common practice in The Philippines and Nigeria–whereas this is not accepted in the Netherlands. The stigma manifested in avoiding behaviours of the public in the streets, public transportation, restaurants or bars, and church. Consequently, some participants hid their identity as COVID-19 HCWs or avoided public spaces and social interactions out of fear of rejection and stigmatization (anticipated stigma).

*Philippine participant 8: “When they see you wearing any hospitals suit, like the scrub suit or identify you as health personnel. They will shy away from you […] When they know that you’re from the hospital. Whether you’re a doctor or a nurse, then you’re a potential threat to their health.”*

*Irish participant 4: “And, you know, normally when you get on the bus or in the public sphere, you just, you know, minding your business and avoiding everybody. Well, everybody’s avoiding you. So that’s it.”*

*Nigerian participant 4: “You dare not wear your uniform in the bus or cab. They look at you, everybody they fear you. Even in Church, they know you are a health care worker, they wave at you, they do not come close.”*

*Nigerian participant 14: “I hide my identity, because if they know where we work, they embarrass us and call us CORO. Saying we should not come near them or sit beside them”*.

Furthermore, some participants experienced friends making bad jokes about the fact they might be a carrier of COVID-19 because of their work. This made them feel hurt and disrespected because they recognized the real fear and anxiety hidden in those jokes. Two Nigerian participants saw their multi-year relationships end because they decided to work in the COVID-19 department. Some participants said they were excluded from sport events, or otherwise made felt unwelcome:

*Dutch participant 2: “I said, we also have a COVID-19 patient in our department. They [sport team members] said: ‘But then you should not be here, because you have been in touch with that patient’. That was when I thought: Yes, well we also have tuberculosis patients, and MRSA patients, and so many others. I really struggled with this.”*

Some participants explained that they understood that persons were trying to protect themselves and their loved ones from infection and that the fear of getting infected by COVID-19 HCWs resulted from a lack of understanding and knowledge on how the virus is transmitted and how the PPE was protecting them well from infection.

*Pakistan participant 6: “The feeling of being left out and discriminated because you work in the hospital and because there’s a high risk for you to infect your family members. I felt the discrimination, but it was fine. They obviously cared for themselves and their family members.”*

Participants from all countries experienced some form of stigmatization in their households and/or residences, which manifested most often in isolation and exclusion. For example, some family-members or flat mates demanded participants [all countries] to take extreme preventative measures before entering the house, which are comparable to those measures advised for COVID-19 patients (e.g. isolation in separate room, avoiding being in the same space (kitchen, bathroom, living room) as flatmates). Some participants [Philippines, Nigeria, Pakistan] were living completely separate from their families, for example in a hotel, a tent outside the house (following lack of finances to afford a hotel), or another apartment. One participant was asked by the flatmates to seek other housing–for which she was rejected because she was a COVID-19 HCW.

*Irish participant 11: “My flatmate came to tell me that I should look for somewhere else to stay. […] I went as far as looking for another apartment. It’s just I was not able to get one, because as soon as they get to know that I am working in the hospital… […] I really feel depressed. […] I was almost going to like… should I quit my job?” […]*

*Pakistan participant 2: “There is one room in our home that we have to wash your hands. Even, even after entering the gate, even before checking the door, and we have to wash our hands, we have to sanitize them. And then immediately after coming inside, we have to wash our clothes or shoes or bag. I mean, I’m not allowed to talk to anyone before I get proper shower and sanitize myself.”*

Some participants from Nigeria, Ireland, and The Philippines experienced stigmatization from their landlords/landladies. For example, a landlady had put hand sanitizers and other disinfectants at the entrance of the building after finding out one participant worked at the COVID-19 department. Another participant experienced hesitation from security to allow entrance in the apartment building. Few participants from Ireland explained they or their colleagues were not selected for apartments because of their profession. Some participants were stigmatized by their neighbours: they were avoided, treated as strangers, or scolded as *“Hey COVID people”*. Few participants had heard about colleagues needing to quit their job to avoid eviction by the landlord.

*Nigerian participant 8: “One of the neighbours called me. […] I had to tell her that I worked in the COVID-19 ward. Since then, if they see me coming, they will hide. It is normal. Even the people working in the hospital who find out I work in the COVID-19 ward, they run away from me.”*

### Self-stigma

At the beginning of the pandemic, an important driver for self-stigma was the lack of awareness and knowledge about COVID-19—particularly the transmissibility of the virus- and the effectiveness and type of PPE to prevent infection. Self-stigma was further facilitated by the unclarity and mistrust in the rapidly changing occupational safety standards and policies (the infection prevention and control (IPC) measures). This resulted in the perception of being at very high risk of infection and consequent fear of infecting others. Consequently, almost all participants self-isolated themselves to avoid meeting family or friends (irrespective of social distance and lockdown measures). The measures taken by the participants to avoid contact with family or friends ranged from keeping their distance, taking extra hygienic measures, and wearing masks inside the house to moving out of the house. Some participants had not seen their parents, children, spouse, or friends for weeks up to months. These self-isolation measures were perceived as highly necessary by participants to prevent them from infecting others.

*Dutch participant 4: “No, I have not seen them. I did not dare.. Because both my parents are a bit older […] I would never forgive myself if I would be the one who is bringing the virus to them. […] I was only after the second vaccination that i hugged my mom. We did not hug for over a year. It was very weird.”*

*Pakistan participant 3: “It’s partly because of me, you know, because I know that it’s a very deadly disease and I have seen people who are very young died in my arms. And I have seen people struggling for their life, you know, for the breaths. So, it’s that. It’s not because of them, it’s because of me. I don’t like to go to other people now because I think that I might be able to infect them because of my duties.”*

### Anticipated stigma

The main driver for anticipated stigma was the fear of social ramifications or judgment [Ireland, Nigeria, Philippines]. Some participants were afraid of the reaction of others (sometimes based on previous experiences), including gossiping and stigmatization, which made them avoid family meetings and social gatherings (anticipated stigma). Furthermore, some participants withheld the information that they worked as COVID-19 HCWs during conversations because they were afraid of stigmatization or tired of previously experienced judgemental or “know better” reactions [Netherlands, Ireland, Nigeria].

*Nigerian participant 13: “So before I left for the COVID-19 centre, I did not tell anybody because of the stigma. […] I began telling people about it when I got used to the thing (pandemic and work) and I have gotten the facts in order to defend myself when people counteract the decision that I took to work on the COVID ward.”*

*Philippine participant 9: “For people who are not frontliners, I already gave up talking to them […] Because we are afraid that… You know, the fear of rejection… They might show their expression they are afraid of us. I spend most of my time with colleagues. […] we accept each other […] we don’t hurt each other*.

### Mental health effects

Except for participants from The Netherlands, participants from Ireland, Nigeria, Pakistan, and The Philippines described the effects of stigma on their mental wellbeing in various ways. They said it caused feelings of depression, sadness, anger, infuriation, demoralization, and discouragement. Others felt they were putting their lives on the line, these incidents made them feel disrespected, unsupported, and isolated within their working environment. Some participants described that the fear of being stigmatized by others resulted in feelings of agitation and loneliness. A few participants were thinking of resigning, or saw other colleagues resign, from their jobs [Ireland and Philippines].

*Nigerian participant 1: “I was unhappy about it. I was somehow depressed too. I feel that what we were doing was to serve our nation and the world and people should appreciate the fact that we put our life on the line to do that. Rather than appreciating, people started stigmatizing.”*

*Philippine participant 4: “Yeah, well, the lowest point when I felt isolated, I almost resigned from what I was doing, because (…) I felt that I cannot last any longer. Not having… Nobody to talk to, nobody to eat with, nobody to sleep with. Nobody to do your things… I felt so isolated. And I thought that I can’t survive that. […] I’m happy that my wife understood what I needed. She’s also Christian, and we just prayed and just told each other if we’re going to get it, we’re going to get it. And she treated me, and my kids treated me as if there was no virus. Since then, during the middle part of last year, that helped a lot. Being a family again, eating together, talking (…) together. And without that, I don’t think I’ll be able to survive. The family supports really.”*

Only a few participants felt less affected by the stigma. They said they were *“understanding where someone is coming from”* when it comes to fear of the disease and consequent stigmatization. Despite experiencing being avoided or excluded by some family members or friends, almost all participants [all countries] also received support from their fellow COVID-19 HCWs, spouse, or other family members or friends. Some Nigerian and Filipino participants sought support in their prayers.

### Less stigma with time

Participants from all countries expressed that they experienced most stigma at the beginning of the pandemic. They said that the lack of knowledge and fear of infection were the main drivers at the beginning of the pandemic of the stigma. Participants said that with time, knowledge and awareness improved, and fear decreased partly due to available vaccination. Self-stigma also decreased with themselves, and family members being vaccinated. Furthermore, with increased knowledge and awareness among the public, other participants felt comfortable to start sharing their work as COVID-19 frontliners.

## Discussion

We conducted a qualitative study on the different aspects of stigma among HCWs directly caring for COVID-19 patients in five countries. Our study results show that participants from all countries experienced various forms of stigma, with similarities in the drivers of stigma but differences in the severity of the manifestations (the extent to which stigma was experienced). The stigmatization of the participants in this study affected their mental wellbeing. Although the stigmatization was perceived to have decreased with time, these study results call for efforts to reduce stigma among COVID-19 HCWs in healthcare facilities and in public.

Stigma related to infectious diseases occurs frequently among HCWs. A systematic review showed that 28% of HCWs experienced stigma and 31% perceived stigma [22], which is comparable to studies reporting on COVID-19 HCWs (proportions ranging from 35%-57%) [23–26]. Participants from our study expressed that the stigma was mainly rooted in the fear of infection. This is supported by a quantitative study from Mexico that showed perceived risk of contagion to be the main predictor of stigmatization [27]. Another study among Nigerian community HCWs also showed that fear of infection resulted avoiding behaviour by community members which led to underutilization of health services [28]. The stigma experienced by participants in our study is in line with other studies [1, 29, 30]. For example, American COVID-19 described being refused entrance at a store or the hairdresser and encountering difficulties in conversations because of others “knowing better” about COVID-19 [31]. Indian COVID-19 HCWs nondisclosed their jobs out of fear of stigmatization and felt guilty of exposing their family members to COVID-19 infection [26]. Similarly, Japanese HCWs also non-disclosed their work as COVID-19 HCW. Furthermore, they were being avoided by the general public, other HCWs at the facility, and isolated themselves from others to avoid stigmatization but also to prevent the transmission of COVID-19 to others [32]. Tunisian HCWs were avoided by friends, neighbours, families, and in social activities and experienced verbal stigmatization on some occasions [33].

This study included participants from five different countries: Ireland, The Netherlands, Nigeria, Pakistan and The Philippines. Although stigma is an universal occurring phenomenon, the manifestations and outcomes are highly contextual and culturally specific [34]. The difference between countries lies within the perception of what is most at stake for those in a specific local social world, which influences self-stigma or stigmatizing behaviours of persons [35]. A systematic review showed that compared to people from high-income countries or with a high-level of education, people from low- and middle-income countries or with lower education are vulnerable populations who may have a greater risk of experiencing stigma [22]. Our study confirms these theories. We showed that COVID-19 stigma is a universal phenomenon that is most often driven by the fear of infection and lack of knowledge and awareness. However, our study also showed that COVID-19 stigma manifested in different forms and severities. For example, self-isolation out of fear of infecting family or friends occurred among participants from all countries. However, the magnitude differed between countries: in The Netherlands and Ireland participants avoided family or friends within the household by separating themselves, whereas some participants from The Philippines, Nigeria and Pakistan moved out of the house. Another example is experienced stigma. Of three countries, participants in The Philippines and Nigeria experienced most stigma in the public which was often facilitated by the practice of wearing uniforms outside health facilities, which is not a common practice in the Netherlands for example. Interestingly, we did not find large differences in experience of stigma within the healthcare facility: participants from all countries were avoided by other non-COVID-19 HCWs. The similarity in experienced stigma in healthcare facilities by COVID-19 HCWs in our study may be explained by the presence of similar stigma facilitators and drivers at the beginning of the pandemic: due to the novelty of the virus and therewith lack of knowledge there was a lack of promotion and professional development for HCWs, a lack of consistent and effective transmission control policies and infrastructure, a lack of authoritarian treatment supervision policies (facilitators), and a lack of knowledge regarding transmission, infectiousness, appropriate transmission control measures, and curability of COVID-19 (drivers of fear-based stigma) [36]. This poses an opportunity for the development of a universal COVID-19 stigma reduction intervention focused on healthcare workers, with slight adaptions to different cultures.

Our study showed that most healthcare workers expressed that their mental wellbeing was affected by the experienced, perceived, or anticipated stigma. A review among African health professionals showed that stigmatization was a common cause of depression, anxiety, poor sleep quality, and psychological distress [37]. Another review showed that social stigma was a predisposing risk factor for the development of burnout among intensive care nurses during the COVID-19 pandemic [38]. Other studies also showed a significant association between stigma and depression, anxiety and psychological distress in COVID-19 HCWs [24, 25, 39]. Poor mental wellbeing negatively impacts job performance and therewith quality of care [8–11]. The association between COVID-19 stigmatization and job performance is partially mediated by anxiety [40]. This calls for the development and implementation of effective stigma reduction tools in healthcare facilities because compromised mental wellbeing of COVID-19 HCWs poses a risk for 1) individual health; 2) quality of care due to reduced job performance; 3) access to care due to the risk of burn-out following compromised well-being.

Rather than inventing the wheel, we should explore the possibility of adjusting existing stigma reduction interventions for healthcare institutions. For example, the Allies approach designed for to reduce tuberculosis stigma in healthcare facilities may be adapted for COVID-19 [36]. Another interesting intervention is “Photovoices” in which participants document and share their experiences through photos: it provides a platform for healthcare workers to come together find support in each other [28]. Furthermore, positive approaches in disseminating information and health education of the general public through involvement of the media and community sensitization may reduce stigma related to COVID-19 and other infectious diseases [33, 37, 41].

We conducted a qualitative study in five countries and were able to reach data saturation in three of them (Ireland, Nigeria, and The Philippines). Because of time constrains and difficulties to recruit participants in The Netherlands and Pakistan we are unsure if we reached data saturation in these countries. Another limitation in this study may have been that some participants did not recognize certain behaviours as stigmatizing. This may underestimate the scope of perceived and experienced stigma. Our study provides valuable insights into the drivers and facilitators of stigma among COVID-19 HCWs, which are useful for developers of stigma reduction interventions.

## Conclusion

Stigmatization of COVID-19 HCWs is driven by fear of infection and lack of awareness, and further facilitated by the rapidly changing infection prevention protocols at the start of the pandemic and the negative association with PPE as depicted in disaster and scary stories by media and films. Stigma manifestations involved all types of stigmas in private life, social life, and in healthcare facilities and compromised mental wellbeing of HCWs. As this may affect HCWs job performance and quality of care, there is a high need to develop interventions to reduce stigma among COVID-19 HCWs.

## Supporting information

S1 File Interview topic list. (PDF)
