# Supplementary material for: Respected but stigmatized: Healthcare workers caring for COVID-19 patients
Source: PLoS One. 2023 Jul 21;18(7):e0288609. doi: 10.1371/journal.pone.0288609 (PMC10361490; doi:10.1371/journal.pone.0288609)
Supplement: S1 File — (PDF) [file pone.0288609.s001.pdf]

Supporting information 1.
Interview Topic List

Introductory questions

1. Could you please state your name, gender, age, and job title?
2. Could you please tell me about your role or function as a healthcare worker, in particular in COVID-19 care? What are your day-to-day activities?

Perceptions of COVID-19

3. What are your perceptions about COVID-19 and its contagiousness?
4. Have you been vaccinated for COVID-19. If yes, why?
5. How do your family/friends/community think or feel about COVID-19?

Working as a COVID-19 healthcare professional

6. Can you describe impressive, challenging, and energizing experiences of caring for COVID-19 patients? How have these experiences effected your mental wellbeing?
7. Could you explain how your working environment has been impacted / changed by COVID-19?
   Probe: relations with colleagues, work pressure, working hours, e.g.

Risk of infection

8. How do you perceive risk for COVID-19 infection at work?
9. Have you ever had the feeling people were afraid to get infected by you?
10. What did you think and feel when you heard colleagues in your hospital were infected?
11. Could you describe how you think non-COVID-19 healthcare workers perceive you and your colleagues? Have you experienced avoidance or exclusion from non-COVID-19 healthcare workers?

Stigma

12. Have you experienced any isolation, exclusion and/or discrimination because you care for COVID-19 patients?
    If yes, explain (by whom / when / how / what did you do?)
13. Can you describe any feelings of shame, fear, and guilt that you experienced towards family, friends, or the community because you care for COVID-19 patients?
    Prompt: When? In front of whom? What did you?
14. Can you describe moments where you doubted or refrained from talking about your work at the COVID-19 department out of fear for stigma?
    Probe: when / how / what did you do?
15. **If vaccinated:** Did the vaccination change these feelings and fears? When? How?

16. How did stigma related to COVID-19 affected your mental health?
    Probe: happiness, stress, depression, anxiety
17. Do you talk about your feelings (such as fear) and experiences with others?
    If yes, what do you discuss and with whom?
    If no, why not?

Family / Work life

18. Has your work as a healthcare professional in the COVID-19 department affected your family / social life?
19. Have you ever been or felt excluded or isolated (temporary / full time) from your family or friends out of fear for infection? If yes, explain.
20. Have you ever excluded or isolated (temporary / full time) yourself from your family or friends out of fear for infection? If yes, explain.

Support

21. Could you describe any source of support that you value in your life, and how this has changed since COVID-19?
        Prompt: family, friends, neighbors, sports, volunteering, other engagement
22. How are you supported at work?
23. Do you desire to receive other/more support? If yes, from whom and in what form?

Mental well-being

Mental well-being before COVID-19

24. Could you describe your mental wellbeing **before** the COVID-19 pandemic?
    a. Did you have any feelings of stress, anxiety, depression or other?
       If yes, explain.

Mental wellbeing since COVID-19

25. Could you describe your mental wellbeing **since** the COVID-19 pandemic?
    a. Did you have any feelings of stress, anxiety, depression or other?
       If yes, explain.
26. Could you describe if and how fear and stigma have affected your mental wellbeing?

Future prospects

27. Following this pandemic, how do you look at the near and far future?
28. Do you have any worries about stigma or your mental wellbeing in the future related to your work as a COVID-19 healthcare worker?
    If yes, explain.

We have reached the end of the interview. I would like to thank you for your time. You have given us very valuable information and insights. Are their any topics that we have not yet discussed during this interview that you would like to point out?

Thank you again.

Sources of inspiration for this topic list:

1. Aughterson et al. (2021). https://www.ncbi.nlm.nih.gov/pmc/articles/PMC7871227/pdf/bmjopen-2020-047353.pdf
   Topic guide: file:///C:/Users/spruijt/Downloads/bmjopen-2020-047353supp001.pdf
2. Fang. 2021. https://www.ncbi.nlm.nih.gov/pmc/articles/PMC7938469/#SP1
   Topic guide: file:///C:/Users/spruijt/Downloads/bmjopen-2020-045048supp001.pdf
3. KNCV. Stigma measurment guidance. https://www.challengetb.org/publications/tools/ua/TB_Stigma_Measurement_Guidance.pdf
